# Cavitation-threshold Determination and Rheological-parameters Estimation of Albumin-stabilized Nanobubbles

**DOI:** 10.1038/s41598-018-25913-8

**Published:** 2018-05-10

**Authors:** Maxime Lafond, Akiko Watanabe, Shin Yoshizawa, Shin-ichiro Umemura, Katsuro Tachibana

**Affiliations:** 1 Graduate School of Biomedical Engineering, Tohoku University, Sendai, Miyagi 980-8579 Japan; 2 Department of Anatomy, Fukuoka University School of Medicine, Fukuoka, Japan; 3 Graduate School of Engineering, Tohoku University, Sendai, Miyagi 980-8579 Japan

## Abstract

Nanobubbles (NBs) are of high interest for ultrasound (US) imaging as contrast agents and therapy as cavitation nuclei. Because of their instability (Laplace pressure bubble catastrophe) and low sensitivity to US, reducing the size of commonly used microbubbles to submicron-size is not trivial. We introduce stabilized NBs in the 100–250-nm size range, manufactured by agitating human serum albumin and perfluoro-propane. These NBs were exposed to 3.34- and 5.39-MHz US, and their sensitivity to US was proven by detecting inertial cavitation. The cavitation-threshold information was used to run a numerical parametric study based on a modified Rayleigh-Plesset equation (with a Newtonian rheology model). The determined values of surface tension ranged from 0 N/m to 0.06 N/m. The corresponding values of dilatational viscosity ranged from 5.10^−10^ Ns/m to 1.10^−9^ Ns/m. These parameters were reported to be 0.6 N/m and 1.10^−8^ Ns/m for the reference microbubble contrast agent. This result suggests the possibility of using albumin as a stabilizer for the nanobubbles that could be maintained in circulation and presenting satisfying US sensitivity, even in the 3–5-MHz range.

## Introduction

Over the past decades, the use of bubbles in the ultrasound (US) diagnostic and therapeutic arsenal has increased^1–5^. They can be used as ultrasound contrast agents (UCA) to enhance the performance of US imaging but also to determine physiological properties, notably blood flow^6–10^. Microbubbles (MBs) are approved worldwide and widely used in characterization of kidneys, the liver, breast, spleen, and pancreas^11^. Bubbles can also be targeted via antibodies to specific molecules in locations of interest^12,13^. For drug delivery, bubbles are ideal carriers for therapeutic material^14,15^. They can cluster in a targeted area and release their payload either naturally or under the action of an external stimulus such as US^16^.

Cavitation is a potent mechanical effect of US and a formidable tool for a large number of applications such as drug delivery^17–21^, sonodynamic therapy^22,23^, hyperthermia^24^, immunotherapy^25,26^, lithotripsy^27^, and histotripsy^28^. However, cavitation usually requires large pressure levels to occur, which may bring about safety concerns. The strength of the bubbles in this case is to act as cavitation nuclei, reducing the required pressure to induce the desired mechanical effect. This also reduces the risk of creating collateral cavitation in untargeted zones. However, cavitation can be obtained only in places reachable by the bubbles in circulation. This is one of the main potential benefits of nanobubbles (NBs) in the therapeutic arsenal. Due to their smaller size, they can access places that are not reachable by MBs. Moreover, they are less prone to clearance and are likely to prolong their blood circulation time^29^. Thus, NBs are of great interest in both imaging and therapeutic purposes^30–33^.

The evidence and manufacturing of NBs seem straightforward as they are just a few times smaller than MBs, which are well characterized and routinely produced. However, there is a physical barrier to the bubble size, which has made the path of NB research a bit more problematic^34^. In the early 1950s, the Epstein-Plesset theory predicted the fast dissolution of NBs^35^. The so-called Laplace pressure bubble catastrophe describes that a bubble in solution will either grow and be removed by buoyancy or shrink and dissolve in the solution. This is shown to be particularly true for small bubbles, as they are unlikely to be thermodynamically stable^36^. However, various encapsulation methods have been developed to reduce the surface tension and prolong NB stability^37,38^. This includes liposomes (lipid bilayer shell including an aqueous core with gas pockets)^39–41^, cavitation seeds acting as gas-pocket traps sur as nanocups^42^ or porous nanoparticles^43^, NBs of lipids, polymers, or protein shells with a gaseous core^44–48^, and nanodroplets (lipid or polymer shell with the specificity of being phase-changing agents)^49^. There are numerous applications for which NBs provide interesting results, notably drug delivery to various targets: tumor^50–58^, nerve^59^, retina^60^, vascular tissues^61^, brain blood barrier^62^. They can also be used to perform gene transfection^63–68^. Finally, they showed interesting features for imaging applications^69–73^ and theragnostic modalities^74–77^.

Cavitation events can be monitored through several modalities, notably cavitation mapping, ultrafast imaging^78^, and passive cavitation detection^79^. Acoustic methods have shown to be potent for determining bubble characteristics^80,81^.

The purpose of this study was to assess the possibility of using albumin-stabilized NBs as cavitation nuclei while monitoring the cavitation activity with a passive cavitation detector (PCD). Also, the cavitation thresholds of the manufactured albumin NBs were measured for two different frequencies. From this information, bubble-oscillation simulations were conducted with a modified Rayleigh-Plesset equation to determine the rheological parameters (dilatational viscosity and surface tension) of the manufactured NBs.

## Material and Methods

### Preparation of NBs

**Figure 1:**
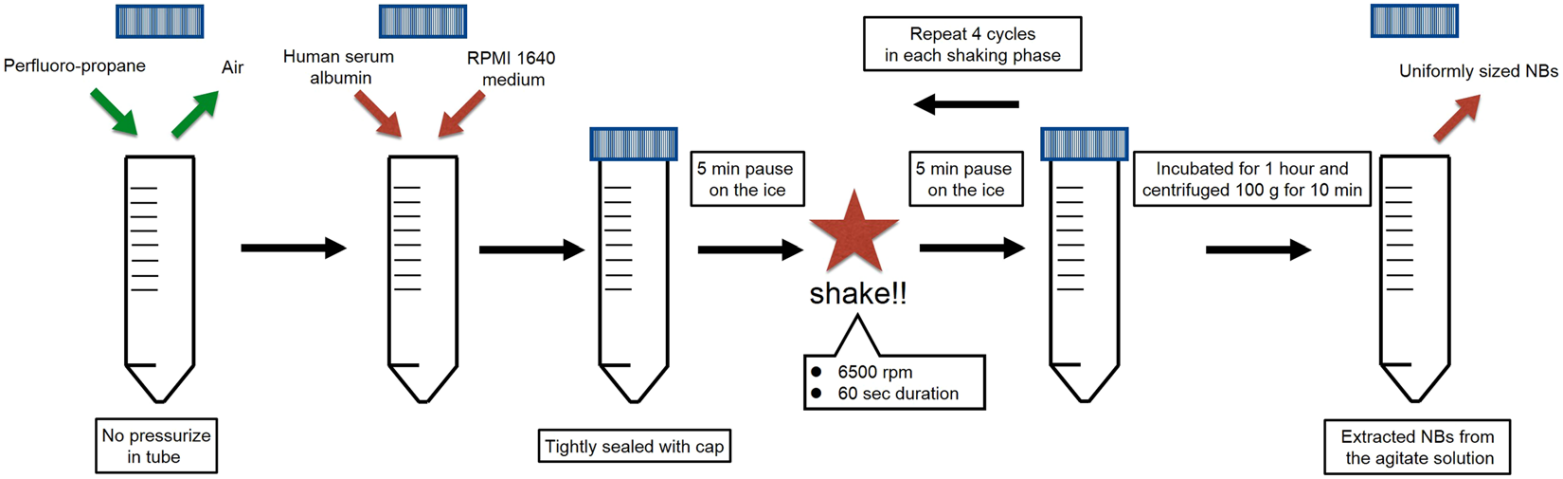
Schematic of the albumin-stabilized nanobubbles manufacturing process.

Human serum albumin NBs were fabricated using a high-speed agitation method. A high-speed agitation device, originally a tissue homogenizer device that provides a three-dimensional multi-directional motion to the fluid container tubes, was used (Precellys Evolution; Bertin Instruments, France). Two materials (both liquid and gas) were placed within custom-made container tubes and agitated at high speeds. The air in a plastic container tube (height, 30 mm, outer diameter, 25 mm) was replaced with 15 mL of perfluoro-propane (C3F8; Takachiho Chemical Industrial Co., Tokyo, Japan) gas using a 23-gauge needle inserted through a cap. As the perfluroro-propane is heavier than air, the air is replaced by perfluroro-and the container is full of propane at ambient pressure. Briefly, a 10-mL sterile solution of 0.06% human serum albumin (fraction V, purity 96%; Aventis Behring L.L.C., IL, USA) in Roswell Park Memorial Institute medium (RPMI 1640; Nacalai tesque, Kyoto, Japan) was added in a C3F8 gas filled container tube. The C3F8 gas and albumin solution in the container were tightly sealed with a cap. This process insures that a reproducible molar amount of gas is used. All the procedures were carried out within a clean bench to avoid nanoparticle contamination. The container tubes were then placed into a high-speed shaking-type tissue homogenizer device previously described and shaken four times at high speed under the following conditions: 6500 rpm, 60-second duration, 5-min pause on the ice between each shaking phase. After finishing all the shaking phases, the samples were incubated at room temperature for 1 hour. To extract uniformly sized NBs from the agitate solution, centrifugation (MX-301; TOMY, Tokyo, Japan) was carried out at 100 g for 10 minutes to separate all MBs and NBs. The NBs included in the solution was then preserved at 4 °C until use. Figure 1 sums up the manufacturing process. Also, an additional video of the sample agitation is linked.

### Bubble characterization

#### Nanoparticle tracking analysis

The particle size of albumin NBs was measured using a nanoparticle tracking analysis (NTA) device (Nanosight LM10; Malvern Instruments Ltd, Worcestershire, UK). The nanoparticle suspension can be illuminated under a 638-nm red laser by using this device. The nanoparticle movement expressed light scattering under Brownian motion and was recorded using a CCD camera (C11440-50B; Hamamatsu Photonics K.K., Shizuoka, Japan). This NTA system automatically detects the center position of nanoparticles and tracks the nanoparticle motion in a two-dimensional plane for calculating the average moving distance under Brownian motion. The particle sizes were estimated by the average moving distance to the Stokes-Einstein equation. The range of particle-size measurement with the NTA method was from 10 to 1000 nm. An NBs suspension of 0.5 mL was injected to the sample chamber of the Nanosight system with a 1.0-mL-volume syringe (Terumo tuberculin syringes SS-01T; Terumo Co., Tokyo, Japan). The image of particle movement under Brownian motion was recorded for 60 seconds at room temperature. Software NTA 3.2 Dev Build 3.2.16 (Malvern Instruments Ltd, Worcestershire, UK) was used for sample-image capturing and data analysis. Three independent experiments were conducted for each sample. The particle size was presented as the average of three measurements.

#### Dynamic light scattering

To measure the physical properties of Sonazoid MBs, a light-scattering measurement system ELSZ - 2000ZS (Otsuka Electronics Co., Osaka, Japan) was used. The measurement principle is dynamic light scattering for observing temporal change or fluctuation of scattered light from Brownian moving particles to estimate the overall size distribution of bubbles. The measurement range of the device was 0.6 nm to 10 μm. All measurements were carried out at room temperature by adding 1 mL of the sample to the glass cell. Repeated measurements were carried out three times for each sample and averaged to determine particle diameter.

There are two main reasons why we used different techniques for the size measurements. Firstly, dynamic light scattering requires a certain bubbles concentration to be accurate (typically more than one billion NBs par mL), criterion that we did not meet with our manufacturing process. On the other hand, nanoparticle tracking analysis is accurate even with lower concentrations. The second reason is that dynamic light scattering relies on a size distribution symmetrically centered around the peak. This was not the case with the albumin-stabilized NBs. In this case nanoparticle tracking analysis is more adapted because it sizes individually each bubble with the bubble concentration determined from the number of bubbles within the field of view.

#### Flow cytometric analysis

To estimate the NBs stability over time, the number of albumin NBs were determined using a flow cytometer (CytoFLEX; Beckman coulter, CA), which was equipped with 405- (violet), 488- (blue), and 638-nm (red) lasers to detect up to 13 fluorescence colors. The cytometer was set up to measure side scatter (SS) from the violet laser for enhanced nanoparticle detection. The violet-SS signal resolution for particle detection was less than 200 nm. To relate violet-SS to particle size, we calibrated the flow cytometer with beads of known size. Polystyrene standard beads (200, 350, and 800 nm; qNano Calibration Particles; Izon sci. Ltd, Christchurch, New Zealand, 500 and 1000 nm; Archimedes Standard polystyrene beads; Malvern Instruments Ltd, Worcestershire, UK) suspended in ultrapure water were measured beforehand with the cytometer. We created the gate based on the size of the standard beads in the range from 200 to 1000 nm for determining the size of our fabricated albumin NBs. The acquired violet-SS signals of the albumin NBs were analyzed using CytExpert analysis software version 2.0 (Beckman Coulter, CA).

### Cavitation measurements

**Figure 2:**
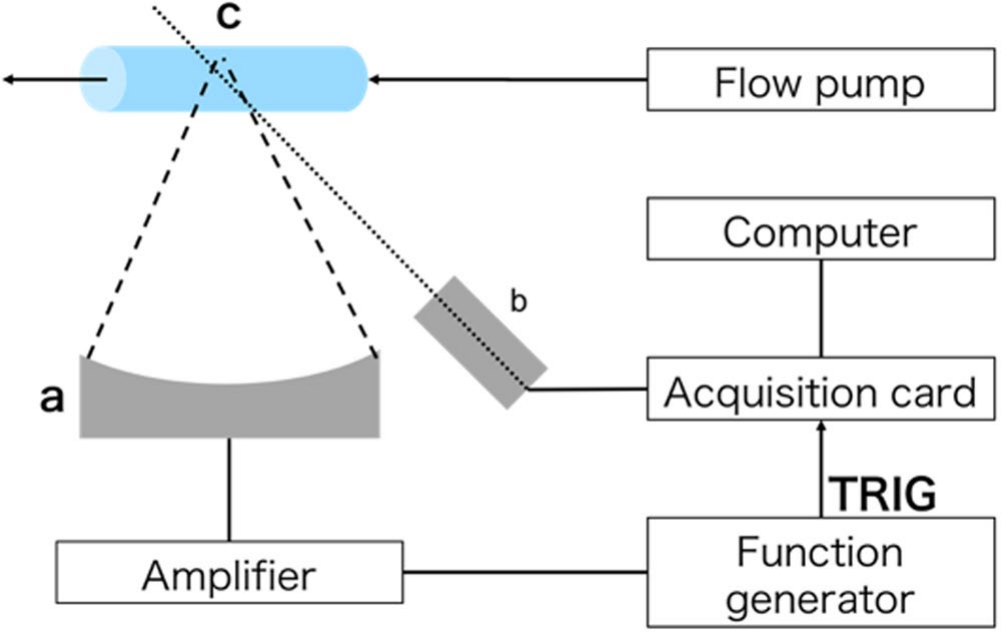
Schematic of experimental setup for cavitation detection. a: Emission transducer. Two different transducers were used: 3.34 and 5.39 MHz. b: Passive cavitation detector. c: Bubbles are circulating at controlled flow speed in acoustically transparent tube placed in focal zone of emission transducer.

As illustrated in Fig. 2, the cavitation-measurement experiment relies on a US emission transducer (a) to stimulate the bubbles circulating at a controlled flow in the focal zone (c), and an aligned in-house PCD (b) receiving the signal from the focal zone. This is then transmitted to an acquisition card for data recording and further processing.

The emission signal consisted of 200-cycle pulses generated by function generation (Wave Factory WF1943, NF Corporation, Yokohama, Japan) spaced by a 50,000-cycle pause between each pulse. The emission signal was then amplified (AG 1006, T&C Power Conversion Inc., Rochester, NY) with a varying gain factor and delivered to the emission transducer. The corresponding focal pressures were measured for shorter pulses (15 cycles) using a needle hydrophone Precision Acoustics SN2203 with a PA14235 preamplifier (Precision Acoustics, Dorchester, UK) for each transducer. The two different piezo-ceramic-focused transducers were used for emission at two different frequencies: 3.34 and 5.39 MHz.

The solutions containing bubbles were injected in circulation in an acoustically transparent tube (Cobalt Polymers, CA, USA; inner diameter, 2.5 mm, thickness, 38 µm) via a syringe mounted on an injection pump (Pump11Elite; Harvard Apparatus, MA). The tube was placed in the focal zone of the emission transducer. This step is particularly important as both the focal area and tube were small. To do so, the water level was first adjusted to coincide with the tube position. Then, on a slightly remote position from the tube (to avoid unnecessary tube deterioration), we adjusted the transducer elevation while emitting on a high duty cycle so that an acoustic fountain forms at the water surface. The tube and focal point were thus on the same elevation at this step. Finally, the position of the fountain was marked with a laser pointer, and the tube was moved to this location while US was off, without changing the elevation. After that, the bubbles were put in circulation in the tube and exposed to US pulses, and the amplifier gain was manually adjusted. The experiment was conducted two times. Once with the albumin NBs, and once with well characterized commercially available ultrasound contrast imaging microbubbles, Sonazoid (Daiichi Sankyo Co Japan) as micro-sized bubble control for the method.

Acoustic noise emitted by the bubbles was recorded for increasing the focal pressure at the two frequencies. The PCD is an in-house polyvinylidenfluorid (PVDF) hydrophone with a wide broadband in the entire frequency range of interest. Signals were acquired using a Red Pitaya StemLab 125-10 acquisition board (Red Pitaya, Ljubljana, Slovenia) and synchronized with the function generator. The cavitation indexes (CI) were calculated in Matlab (The Mathworks, Inc., Natick, MA) from the average value of the Fourier transform between 2 and 20 MHz:

1 $$CI=\frac{1}{N}\sum _{f=2MHz}^{20\,MHz}20lo{g}_{10}\Vert S(f)\Vert ,\,$$

where N designates the number of points in the frequency interval considered and *S*(*f* ) is the Fourier transform of the received signal *S*(*t*) calculated by the *fft* function.

### Numerical study

We used a Rayleigh-Plesset-type equation to model the oscillation of a bubble under the US excitation ${p}_{A}(t)$ with the Newtonian rheology parameters of the bubbles. This rheological model takes into account a viscous encapsulation of the bubble, characterized by the surface dilatational viscosity ${\kappa }^{S}$ and surface tension $\gamma$ the bubble oscillation can be expressed as

2 $$\rho (R\ddot{R}+\frac{3}{2}{\dot{R}}^{2})={P}_{G0}{(\frac{{R}_{0}}{R})}^{3k}-4\mu \frac{\dot{R}}{R}-\frac{4{\kappa }^{S}\dot{R}}{{R}^{2}}-\frac{2\gamma }{R}-{P}_{0}+{p}_{A}(t),$$

where $\rho$, ${P}_{0}$, and µ are the density, hydrostatic pressure, and viscosity of the surrounding medium, respectively, R is the bubble radius, $\dot{R}$ and $\ddot{R}$ its temporal first and second derivative (thus the bubble wall speed and acceleration), and $k$ is the polytropic exponent of the gas inside the bubble: $k=1$ corresponds to isothermal gas behaviour. For the Sonazoid MBs, this exponent is very small: $k=1.006$^81^. As both the Sonazoid MBs and manufactured NBs were observed to be stable for at least 2 hours, we can assume a pressure equilibrium ensuring bubble stability as an initial condition. Thus, the initial pressure inside a bubble ${P}_{G0}$ can be expressed as

3 $${P}_{G0}={P}_{0}+2\frac{\gamma }{{R}_{0}}.$$

Equation (2) is solved using a fourth-order Runge-Kutta method implemented in Matlab with the *ode45* function. We determine if the bubble reached the cavitation threshold by assessing the inward bubble wall speed: the inertial cavitation threshold is considered reached for inward speeds above 340 m/s.

The bubble oscillation was modeled for a large range of the two investigated rheological parameters ${\kappa }^{S}$ and $\gamma$. For each set of values, we attributed a score depending on how this set corresponds to the measured pressures. A score of 1 indicates that this set of values allows cavitation inside the measured pressure interval for one frequency only. A score of 0 indicates that the calculated threshold is outside the measured interval for both configurations. If the threshold from simulation is inside the measured interval for the two frequencies, a score of 2 is attributed. A score of −1 indicates aberrant results. Thus, the region with a score of 2 gives the range of rheological parameters of the studied bubbles. This parametric investigation was run twice; once on a very wide parameter range with a rough grid and once in the resulting region of interest with a more refined grid.

## Results

### Bubble-size measurements

**Figure 3:**
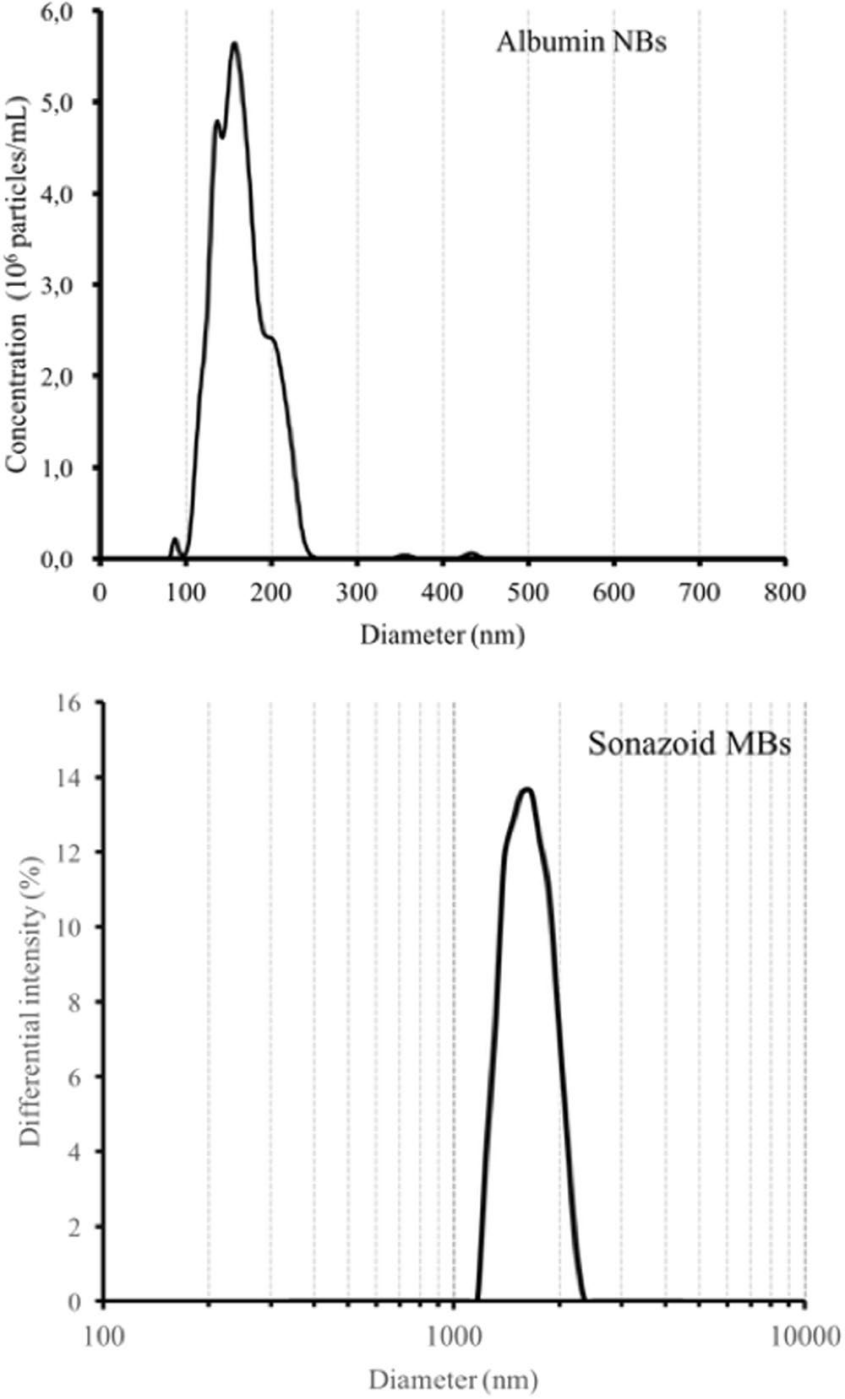
Measurement of bubble size for albumin NBs (top) and Sonazoid MBs (bottom).

**Figure 4:**
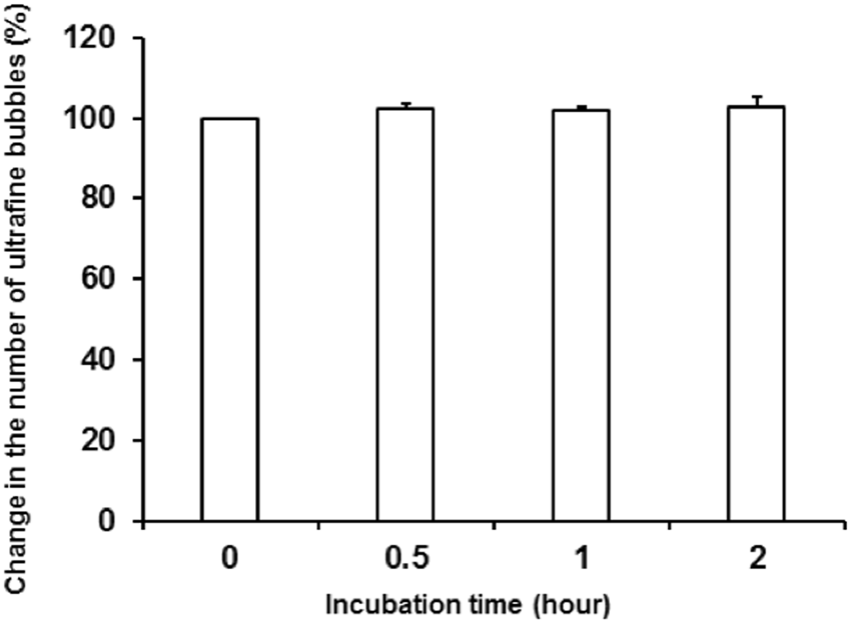
Albumin NBs stability over time. Total number of NBs remained unchanged for at least 2 hours, which was approximate duration for cavitation experiment. Errors bars represent the standard deviation with N = 3. Student t-test does not indicate a significant change in the percentage of bubbles compared to their initial number (at t = 0).

Figure 3 presents the size distribution of the albumin NBs and the Sonazoid MBs. From these measurements, we consider 165-nm bubbles in simulations for albumin NBs. The mean size of the Sonazoid MBs was measured at 1.9 µm. This is slightly at odds with the expected size. In fact, the size of the Sonazoid MBs was indicated to be in the 2.3–2.9 µm range according to the manufacturer’s information^82^. Fig. 4 shows the stability over time of the albumin-stabilized NBs on two hours (approximate duration of the experiment).

### Cavitation-threshold determination

**Figure 5:**
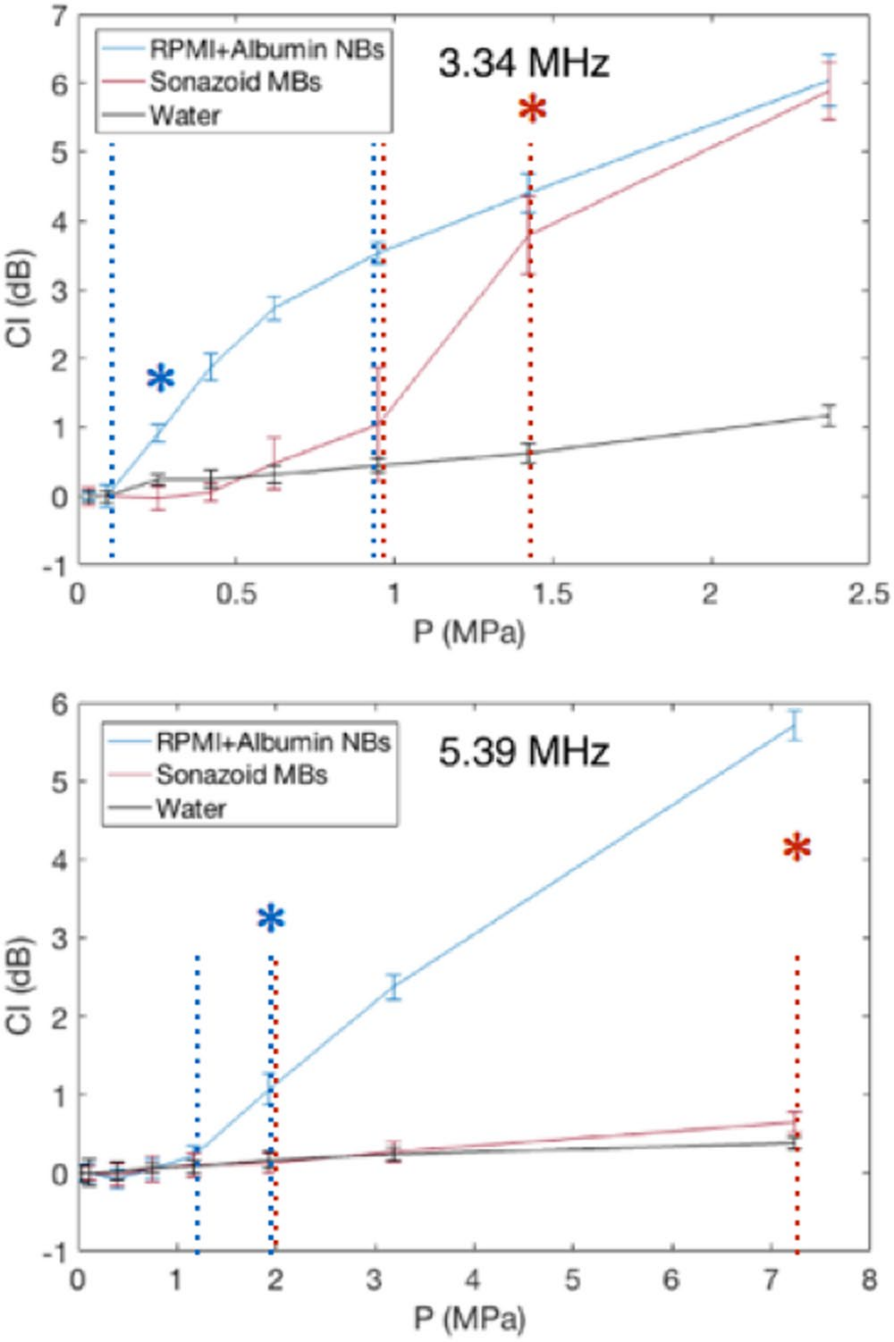
Cavitation-threshold measurements for two types of bubbles at frequencies of 3.34 MHz (top) and 5.39 MHz (bottom). Error bars represent standard deviation of CI over 10 measurements for each pressure level. The blue and red dashed lines (for Albumin NBs and Sonazoid MBs, respectively) represents the pressure interval that is assumed to comprise the cavitation threshold. The asterisk states a statistically the lower pressure parameter with a significant difference relatively to the CI in degassed water (p < 0.01) (it is not displayed for higher pressures for figure clarity).

Figure 5 illustrates the cavitation index (CI) measured from the received signal for various focal pressures for the Sonazoid MBs, albumin NBs, and in reference degassed water. The elevation of the CI level above the reference values (water) provided evidence of cavitation presence. This was less clear for the Sonazoid case in the 5.39 MHz configuration. A significant elevation was still detected for a pressure of 7.2 MPa. The elevation was also substantial at 3.1 MPa without being very clear. We thus consider 1.9 MPa to be our considered pressure below the cavitation threshold.

For each configuration, we selected a pressure value above and one below the assumed cavitation threshold. Those pressure are indicated by the dashed lines. The statistical significance (p < 0.01 with a Student t-test) of the difference of CI with degassed water is indicated by the asterisk. One could notice that is some cases (Albumin NBs at 3.34 MHz and Sonazoid MBs at 5.39 MHz), the chosen pressure values do not strictly correspond to just before and after significance as it is the case in the other conditions. In the first, it is because the CI elevation according to the excitation pressure is very steep. We hypothesized that it was due to the strong echogenicity of these NBs and we preferred to take a higher value of pressure to be sure to comprise the cavitation threshold in the interval. In the other case (Sonazoid MBs at 5.39 MHz), the CI value at 3.2 MPa was not significantly different from the degassed pressure value, but with a p-value of close to 0.1. Thus, as previously, we preferred taking some margin to be sure to comprise the cavitation threshold in the chosen interval. The pressure values in MPa are summarized in Table 1.

**Table 1:** Pressure parameters used in simulations from cavitation measurements.

Configuration	P above (3.34 MHz)	P below (3.34 MHz)	P above (5.39 MHz)	P below (5.39 MHz)
Sonazoid MBs	1.4	0.9	7.2	1.9
albumin NBs	1.0	0.1	1.9	1.2

### Rheological-parameter determination

**Figure 6:**
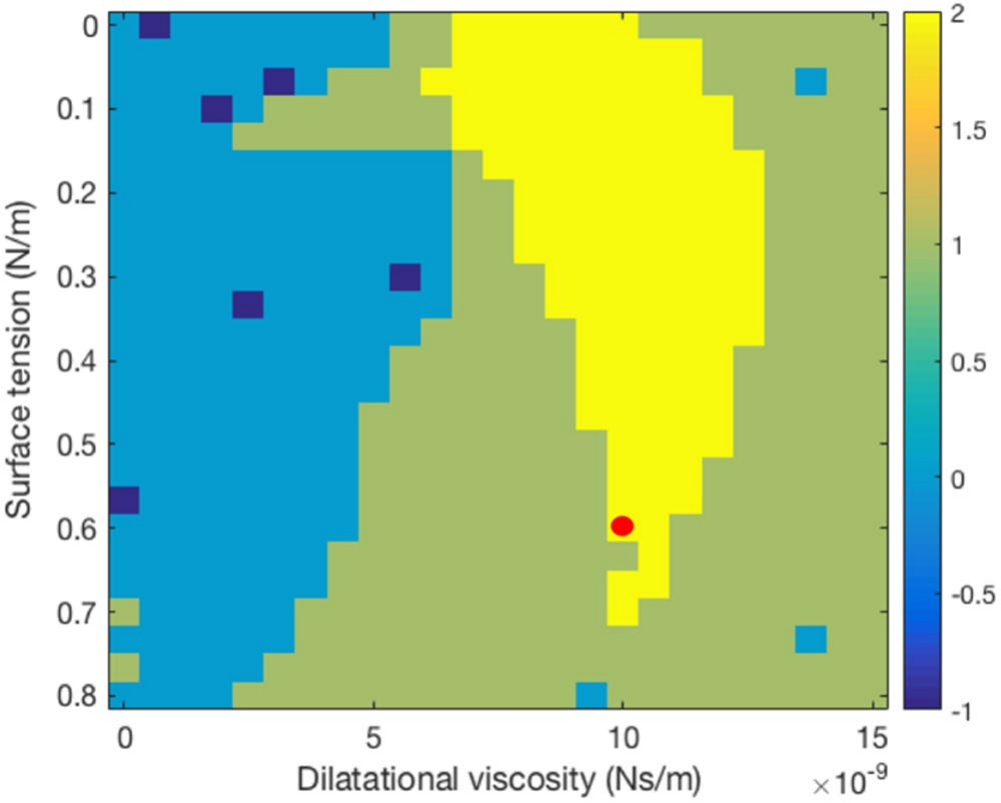
Determination of rheological parameters of Sonazoid MBs. Region in yellow corresponds to plausible parameter sets. Red dot corresponds to rheological parameters found in literature.

**Figure 7:**
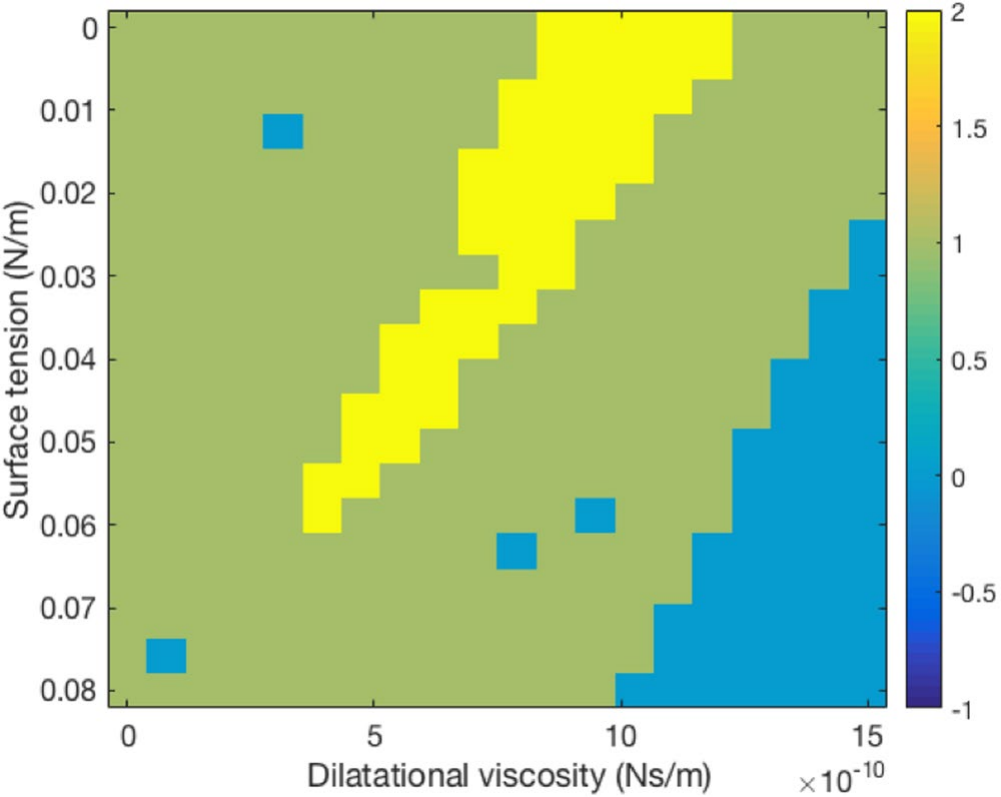
Determination of rheological parameters of albumin NBs. Region in yellow corresponds to plausible parameter sets.

Figure 6 illustrates the rheological-parameter determination for the Sonazoid MBs. The region in yellow corresponds to plausible parameter sets (scored 2). This region includes the parameters set of 0.6 N/m and 1.10^−8^ Ns/m, which has been reported for Sonazoid MBs^81^. Fig. 7 illustrates the rheological parameters estimated for the manufactured albumin NBs. The plausible parameters are located on a thin conical region ranging from 0 N/m to 0.06 N/m in surface tension. The corresponding values of dilatational viscosity ranged from 5.10^−10^ Ns/m to 1.10^−9^ Ns/m.

## Discussion

In this study, we showed that albumin NBs can be used effectively as cavitation nuclei, with inertial cavitation thresholds under 2 MPa. We also showed that information on cavitation thresholds can be used to investigate the rheological parameters of these NBs through bubble-oscillation simulations.

The Sonazoid MBs, the parameters of which have been reported, were used as a test case. The potential rheological parameters were found in a relatively wide range, including the reference value from the literature^81^. Applying the same method to the albumin NBs permitted estimation of their rheological parameters. These are located in a conical region ranging from 0 N/m to 0.06 N/m in surface tension. The corresponding values of dilatational viscosity ranged from 5.10^−10^ Ns/m to 1.10^−9^ Ns/m. These values are both orders of magnitude below those of the Sonazoid. As a comparison, the surface tension at a water-air interface is 0.072 N/m and in an aqueous albumin solution between 0.035 and 0.072 N/m depending of the albumin concentration^83^. Moreover, the cavitation measurements prove that the dilatational viscosity is a key parameter, as no parameters set was found plausible with a null dilatational viscosity. The experiments we conducted did not provide precise information on the manufactured bubbles’ structure. Nevertheless, we can hypothesize that these are either comparable with extremely soft-shelled NBs (as we modelled) or gas vesicles (the albumin trapping the gas during shaking). The observations and possibility to simulate the NB behavior with a rheological model tend to confirm the hypothesis that they behave as NBs. The most likely hypothesis is that albumin acts as a surfactant rather than a shell such as the Sonazoid lipid hard shell. This would prove the possibility to stabilize NBs with only viscous surfactant. In the albumin-stabilized NBs stability results presented in this study, we displayed only their stability along the approximate two hours of the experiment. However, to be pertinent in a clinical context, the produced NBs have to remain stable longer. Additional analysis on the storage stability of these nanobubbles remains to be done. As the shaker to produce the bubbles is quite standard, affordable and possible to miniaturize, it is conceivable that in future developments, the bubbles can be produced routinely, directly in the clinical environment by shaking prepared samples.

Albumin NBs present a much lower dilatational viscosity and are much softer than Sonazoid-shelled MBs. This can explain why albumin NBs can be kept stable for hours and even injected in circulating flows but maintaining an impressive sensibility to US and a low cavitation threshold. The lowering of sensitivity to US has been reported to be one of the major issues of the submicron transition^38^. One of the main advantages of albumin NBs is that the sensitivity to US remains excellent, even for standard therapy and imaging frequency range (3–5 MHz). These frequencies are relevant of imaging and therapy using NBs^48^. This range permits the preservation of the penetration depth and low thermal effects. The sensitivity and noise emission from inertial cavitation was even greater than with the Sonazoid MBs.

As the pressure values we selected become closer to the actual threshold, the accuracy of the suggested method to determine the rheological parameters would improve. However, the bubbles present a relatively wide size distribution. Consequently, the threshold in this case is not a single pressure value but rather a pressure range. The pressure range can be quite wide: in the case of the albumin NBs, the interval ranged from 0.1 to 1.0 MPa. Although the cavitation threshold seemed to be reached even for very low amplitudes, we hypothesized that these early cavitating bubbles may be the upper limit of the size distribution. Consequently, the two values we selected for simulations were the pressure value before this threshold and an upper value after that the curve slope becomes gentler. It should be noted that we modeled the oscillation of single bubbles, and a few factors affecting their growth were not taken into account. Notably, rectified diffusion (higher gas inflow than gas outflow during oscillation phases)^84^, coalescence, and Ostwald ripening (growth by diffusion: the larger bubbles “vampirize” the smaller)^85^, were not taken into account. Nevertheless, it was observed in simulations that collapses occur very early in the oscillation process for these bubble parameters, within the first cycle, discarding rectified diffusion as a leading growth mechanism in this particular case. Ostwald ripening and coalescence are much more delicate to evaluate. However, the bubble-size distribution was measured to be stable for at least 2 hours (duration of the sonication experiment), and no change in the cavitation activities were reported while using longer pulses (400 and 600 cycles instead of 200, data not shown). This suggests that the presence of US did not affect coalescence and Ostwald ripening. Furthermore, it was shown that NB clusters (which would possibly be the case under an acoustic field due to Bjerknes forces)^86,87^ can exhibit a shielding effect against Ostwald ripening^85,88^. Finally, another factor that could affect the results is that we only considered the mechanical stress of US. However, thermal effects occurring locally during the pulses may result in a denaturation of the albumin constituting the NBs. This denaturation might change the tension surface of the albumin^89^; thus, the cavitation threshold.

## Conclusion

We demonstrated that albumin NBs in the 100–250-nm range were sensitive to US and could be used as efficient cavitation nuclei in the 3–5-MHz range at least. Their inertial cavitation threshold was lower than the commercially available Sonazoid micro-sized UCA. Simulations were conducted with a modified Rayleigh-Plesset equation according to Newtonian rheology. The determined values of surface tension and dilatational viscosity ranged from 0 N/m to 0.06 N/m in surface tension. The corresponding values of dilatational viscosity ranged from 5.10^−10^ Ns/m to 0 1.10^−9^ Ns/m. These values were 0.6 N/m and 1.10^−8^ Ns/m for the reference Sonazoid MBs. This suggests that NBs can be stabilized using surfactant only, providing high US sensitivity.

## Electronic supplementary material

Video
